# Human Factors and Device Engineering in OTC Intranasal Drug Delivery: GentleMist Technology™

**DOI:** 10.3390/pharmaceutics18091128

**Published:** 2026-09-08

**Authors:** César Alas-Pineda, Dennis J. Pavón-Varela, Kristhel Gaitán-Zambrano, Carlos Coto-Tejeda, Jhacely Medina-Mejía, Nelly Andrews Interiano, Gustavo Ferrer

**Affiliations:** 1 Department of Research and Development, Moxie Health Group, Hallandale Beach, FL 33009, USA; dpavon@moxie-health.com (D.J.P.-V.); kgaitan@moxie-health.com (K.G.-Z.); ccoto@moxie-health.com (C.C.-T.); jmedina@moxie-health.com (J.M.-M.); nandrews@moxie-health.com (N.A.I.); 2 Department of Pulmonary and Critical Care Medicine, Aventura Hospital and Medical Center, Aventura, FL 33180, USA; gferrer@pulmonary-institute.com

**Keywords:** intranasal drug delivery, nasal spray, nasal biopharmaceutics, computational fluid dynamics, human factors engineering, over-the-counter drug products, regional deposition, drug–device combination

## Abstract

Intranasal drug delivery is noninvasive, rapid-acting, and well suited to self-administration, yet the real-world performance of nasal sprays depends on the interaction among formulation, device mechanics, nasal anatomy, and user technique rather than on the active ingredient alone. This narrative review examines how aerosol science, regional targeting, and human factors jointly shape intranasal performance, using GentleMist Technology™ as a representative example of emerging device-centered development. Computational fluid dynamics (CFD) and anatomically informed in vitro models indicate that device geometry, plume characteristics, and administration angle can significantly alter where a dose is deposited, while human factors research identifies priming, positioning, and angulation as key determinants of successful over-the-counter (OTC) use. We summarize early feasibility data suggesting that angle-optimized administration may increase posterior nasopharyngeal delivery and that essential tasks are feasible for lay users, with priming emerging as a specific usability vulnerability. We also review preliminary clinical evidence on a chlorpheniramine maleate (CPM)-based intranasal formulation in allergic rhinitis and viral upper respiratory illness. Although the strongest device-specific findings remain preliminary and require independent replication, they support an emerging shift from a formulation-centered model toward an integrated device–formulation–user paradigm.

## 1. Introduction

Intranasal drug delivery has emerged as a strategically important route in contemporary pharmaceutics. The nasal cavity provides a highly vascularized epithelial surface, avoids hepatic first-pass metabolism, allows rapid onset of action, and supports self-administration without needles or specialized equipment [1,2,3]. Historically, nasal products were developed primarily for topical treatment of rhinitis and congestion, but the field has expanded substantially to include rescue medicines, vaccines, systemic therapies, biologics, and selected nose-to-brain approaches [1,2,3]. This expansion has made it increasingly clear that intranasal products should not be understood simply as formulations packaged in a spray bottle. Their performance depends on the convergence of formulation science, device engineering, anatomy, and user behavior.

Despite substantial advances in nasal biopharmaceutics, a significant translational gap remains. Most intranasal products are evaluated primarily using emitted dose and conventional spray metrics, whereas far fewer development pathways establish mechanistic links between spray generation, regional deposition, mucosal retention, patient use, and ultimately therapeutic efficacy [4]. This challenge is further amplified by increasing recognition that distinct regions of the nasal cavity exhibit specialized anatomical and physiological functions. The upper nasal space, for example, has increasing relevance for systemic delivery, mucosal vaccination, and nose-to-brain applications because deposition in that region may result in different absorption and residence characteristics than deposition in the lower anterior cavity [3,5].

From a practical standpoint, the success of intranasal therapy is also strongly influenced by how the patient actually uses the product. Real-world technique is variable, and many users do not follow optimal positioning, priming, or administration steps. Community-based evidence has shown widespread use, abuse, and misuse of nasal medications, particularly in contexts where products are readily available without prescription oversight [6]. These issues are particularly important in OTC products, where correct and repeatable use depends largely on device intuitiveness, instructional clarity, and user experience.

Against this background, an intranasal drug delivery system like GentleMist Technology™ should be understood not simply as an incremental product innovation, but as part of the broader evolution toward engineered intranasal delivery platforms that integrate formulation science, device engineering, and human factors to optimize therapeutic performance. The specific knowledge gap this review addresses is the limited integration, in the existing literature, of device engineering, regional deposition, and human-factors evidence into a single appraisal framework, and the tendency to present device-specific, formulation-specific, and developer-associated evidence in close proximity without distinguishing their differing strength. The aim of this structured narrative review is to examine how aerosol mechanics, regional deposition, and human factors influence OTC intranasal product performance. GentleMist Technology™ is used as a single, traceable illustrative platform because it is one of the few OTC-oriented systems for which device-specific in vitro, human-factors, and formulation-adjacent clinical data have all been published, allowing the same appraisal framework to be applied across evidence types. Chlorpheniramine maleate (CPM) recurs as the model drug in the platform-associated clinical literature because it is a well-characterized, first-generation antihistamine with an established OTC safety profile and existing intranasal formulation and clinical data, making it a convenient probe compound for evaluating delivery rather than a novel active under investigation. We distinguish throughout between intended design characteristics, device-specific evidence, formulation-specific clinical findings, and proposed applications that remain untested, interpreting evidence according to study design, device specificity, comparator selection, and replication status.

## 2. Methods

We conducted a structured narrative review of intranasal device engineering, regional deposition, and human factors in over-the-counter drug delivery, reported in accordance with the SANRA (Scale for the Assessment of Narrative Review Articles) quality criteria. PubMed was searched from database inception through 3 July 2026. Search strings combined device and delivery terms with performance and human-factors terms using Boolean operators, for example: (“intranasal” OR “nasal spray” OR “nasal drug delivery”) AND (“device” OR “nozzle” OR “plume geometry” OR “droplet size” OR “computational fluid dynamics” OR “CFD” OR “regional deposition” OR “administration angle”) AND (“human factors” OR “usability” OR “priming” OR “self-administration” OR “adherence”); a parallel string paired the intranasal terms with (“chlorpheniramine” OR “CPM” OR “xylitol”) to capture formulation-specific clinical evidence. Reference lists of relevant articles were screened to identify additional studies. Regulatory guidance and official technical sources were consulted when relevant to human-factors principles or device characteristics. Records were screened by title and abstract and then at full text against predefined criteria. We included peer-reviewed computational, in vitro, human-factors, and clinical studies relevant to nasal-spray performance or self-administration, published in English. We excluded studies without a nasal-delivery focus, conference abstracts without full data, and opinion pieces without primary or synthesized evidence; non-peer-reviewed technical materials describing the GentleMist Technology™ (Hallandale Beach, FL, USA) platform were retained only to describe intended device characteristics and were not treated as evidence of comparative performance or clinical benefit. Approximately 680 records were identified, 512 were screened after de-duplication, and 30 met the inclusion criteria and informed this review; a study-selection flow diagram is provided in Figure S1 (Supplementary Materials).

We included peer-reviewed computational, in vitro, human-factors, and clinical studies relevant to nasal-spray performance or self-administration. Evidence was categorized as general intranasal-delivery evidence, GentleMist-specific device evidence, formulation-specific evidence that did not isolate the device contribution, or proposed applications without direct platform-specific data. Given the heterogeneity of study designs, experimental models, comparators, and outcomes, findings were synthesized narratively without meta-analysis or formal certainty grading. Non-peer-reviewed technical materials were used only to describe intended device characteristics and were not considered evidence of comparative performance or clinical benefit.

## 3. Evolution of Intranasal Drug Delivery

The history of intranasal administration reflects a gradual shift from simple liquid instillation toward more controlled and purpose-built delivery systems. Early nasal drops were easy to formulate but provided limited dose precision and highly variable administration. Metered pump sprays represented a major advance by improving convenience and dose reproducibility, and they became the dominant platform for saline products, decongestants, antihistamines, and intranasal corticosteroids. However, conventional spray pumps largely evolved around the concept of emitting a reproducible dose, not necessarily around the goal of optimizing regional deposition or improving patient experience.

As the field matured, it became apparent that emitted dose alone could not explain differences in effectiveness between nasal products. Device-related factors such as plume geometry, droplet size distribution, spray velocity, insertion depth, and nozzle orientation influence whether the formulation deposits anteriorly, drains into the throat, or reaches more posterior and clinically relevant mucosal regions [1,7,8,9,10,11]. This realization has driven interest in more specialized delivery systems, including bidirectional platforms, upper-nasal-space targeting devices, and computationally guided administration strategies [2,12].

The broader evolution of intranasal drug delivery has therefore shifted from a formulation-centered to a performance-centered approach, in which therapeutic success is determined by the integrated interaction of formulation, device design, patient anatomy, and user behavior. Within this evolving framework, the value of a nasal delivery device is no longer defined solely by its ability to deliver a metered dose, but also by its capacity to facilitate reliable administration, generate a spray plume appropriate for the intended target region, minimize patient discomfort, and perform consistently across diverse users and anatomical variations. GentleMist Technology has been developed within this broader device-centered framework. Whether it provides more reproducible regional delivery or a more acceptable administration experience than defined comparator devices remains to be established.

Positioned within this field, GentleMist Technology™ occupies an intermediate technological space: it is more engineering-oriented than a conventional metered-dose pump spray, which is optimized for reproducible emitted dose rather than region-selective deposition, yet it is a single-actuation, self-administered OTC device rather than a specialized targeting system such as a bidirectional breath-powered device or a dedicated upper-nasal-space delivery platform. Its distinguishing proposition is therefore not a new anatomical target but the integration of plume and angle control with human-factors-oriented, comfort-focused design in a format intended for unsupervised OTC use; whether this integration yields more reproducible regional delivery than defined comparator devices remains to be established.

### GentleMist Technology™ as a Device Platform

GentleMist Technology™ is positioned as an intranasal drug delivery platform designed to address two persistent limitations of conventional nasal sprays: administration-related discomfort and variability in regional drug deposition. Rather than functioning solely as a metered-dose spray device, the platform integrates ergonomic design, nozzle engineering, and controlled plume formation to support comfortable administration, dosing reproducibility, and region-directed delivery. These features define the engineering rationale of the platform, although their comparative performance and clinical relevance require confirmation in appropriately controlled studies.

A recently published proof-of-concept study identifies fine-mist generation and controlled dispersion as central design aims [13]. The platform incorporates a tapered nozzle configuration intended to create a broad, swirling atomization pattern designed to distribute medication more evenly across the nasal mucosa while reducing excessive impact, localized irritation, and runoff. These are stated design objectives rather than experimentally demonstrated performance benefits; their comparative validation is considered separately below. In this sense, the platform is framed not only as a drug delivery mechanism, but also as a means of improving the intended administration experience.

The translational importance of this framing is that GentleMist™ emerges as more than a comfort-oriented spray modification. It is better understood as a device platform whose intended value lies in the integration of plume design, patient tolerability, administration geometry, and controlled dosing. This broader identity is particularly relevant in self-administered and over-the-counter settings, where the success of a nasal product depends not only on the emitted dose, but also on whether the device is intuitive, acceptable, and reproducible in real-world use.

## 4. Clinical and Technical Limitations of Conventional Nasal Sprays

Conventional nasal sprays remain constrained by both technical and behavioral limitations. A central technical limitation is that much of the emitted formulation may deposit in the anterior cavity or exit via runoff or throat drainage rather than reaching target mucosal surfaces [1,7,8,9,14]. Comparative in vitro work has shown that nozzle design and administration angle can meaningfully alter delivery efficiency, confirming that the same nominal dose may produce different regional deposition patterns depending on the device and how it is used [10,11].

A second major limitation is anatomical variability. Recent computational and anatomical studies indicate that the vestibule, turbinate geometry, septal configuration, and other structural differences can substantially alter local airflow and spray deposition [9,14,15]. Even physiologic fluctuations such as the natural nasal cycle may change deposition patterns, making regional drug delivery a moving target rather than a fixed mechanical process [16]. This is highly relevant for products that seek to target posterior regions, the nasopharynx, or the upper nasal space.

A third limitation is the administration experience itself. Many patients report burning, dripping, bitterness, throat drainage, or other unpleasant post-spray sensations, and these sensory attributes can reduce acceptance and long-term use [4,17]. A recent systematic review and meta-analysis in allergic rhinitis found that adherence to intranasal corticosteroids is frequently suboptimal and that sensory attributes and adverse effects are among the contributors to poor adherence [17]. These findings reinforce the idea that tolerability is not merely a marketing issue, but a clinically relevant feature of product design.

Finally, misuse and poor technique are common. A real-life community pharmacy study documented substantial use, abuse, and misuse of topical nasal medications [6]. Similarly, educational interventions in allergic rhinitis have shown that targeted pharmacist-led instruction can improve symptom control, underscoring how much product performance may depend on user understanding and technique rather than product composition alone, although the same trial found no significant improvement in medication adherence, knowledge, or quality of life, so this effect appears to be partial rather than comprehensive [18].

## 5. Aerosol Science and Fluid Dynamics as Determinants of Intranasal Performance

A rigorous understanding of intranasal performance requires attention to aerosol science. Once a formulation leaves the actuator, its therapeutic relevance is shaped by droplet size distribution, plume angle, spray momentum, breakup behavior, and the interaction of droplets with inspiratory airflow and nasal structures [1,7,8,9,10]. These factors determine where the spray deposits, how broadly it spreads, whether it reaches posterior regions, and whether it is likely to provoke discomfort or runoff.

Recent CFD-based studies have strengthened the rationale for design-guided intranasal drug delivery. Model-based work has shown that administration angle and device parameters can significantly alter nasopharyngeal targeting [7,8]. Mechanics-guided parametric modeling has further suggested that droplet size, cone angle, and formulation density influence the likelihood of reaching posterior target sites [7]. Additional computational studies have shown that vestibule anatomy and other structural features can affect spray transport and deposition outcomes [8,14,15]. Together, these findings indicate that intranasal drug delivery is highly geometry-sensitive.

Experimental evidence complements these computational results. Comparative in vitro studies using 3D-printed nasal cavity models have demonstrated that different nozzle designs and administration strategies produce different delivery efficiencies and deposition profiles [10,11]. Patient-experience studies evaluating computationally guided administration protocols have also suggested that a shallower-angle technique may be both more comfortable and more favorable for deeper penetration than a more vertical orientation [13].

These findings have several implications for next-generation platforms. First, the emitted spray should be thought of as a region-targeting system, not simply a liquid plume. Second, device optimization and administration instructions should be developed together rather than separately. Third, patient comfort may itself be partly explained by fluid-mechanical behavior, including impact pressure and local plume dynamics. GentleMist Technology™ is therefore particularly relevant because it sits at the intersection of atomization control, geometry-guided delivery, and the engineering of a gentler administration experience.

### 5.1. GentleMist Technology™ Design Features and Proposed Performance Advantages

**Figure 1:**
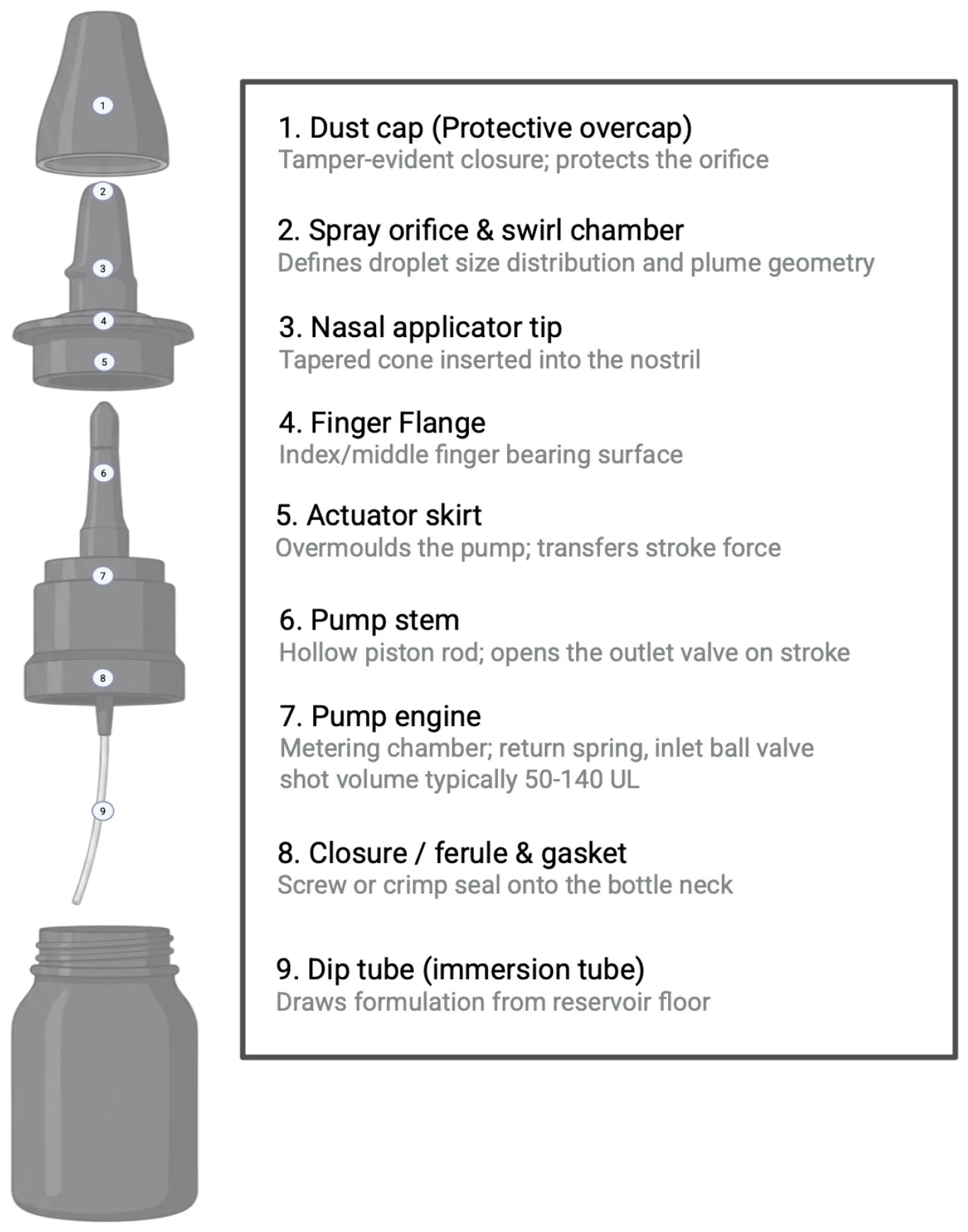
Exploded schematic of the GentleMist Technology™ nasal spray device showing its principal structural components, including the dust cap, spray orifice and swirl chamber, nasal applicator tip, finger flange, actuator skirt, pump stem, pump engine, closure/ferrule and gasket, and dip tube.

GentleMist Technology™ is characterized by several design features (Figure 1) that collectively suggest a distinct intranasal delivery strategy [13,19,20]. These include soft-mist generation, a tapered nozzle system, open-angle swirling atomization, controlled dispersion, and a locking mechanism intended to reduce accidental actuation [19,20]. Together, these elements position the device as an engineered alternative to conventional high-velocity nasal sprays.

**Figure 2:**
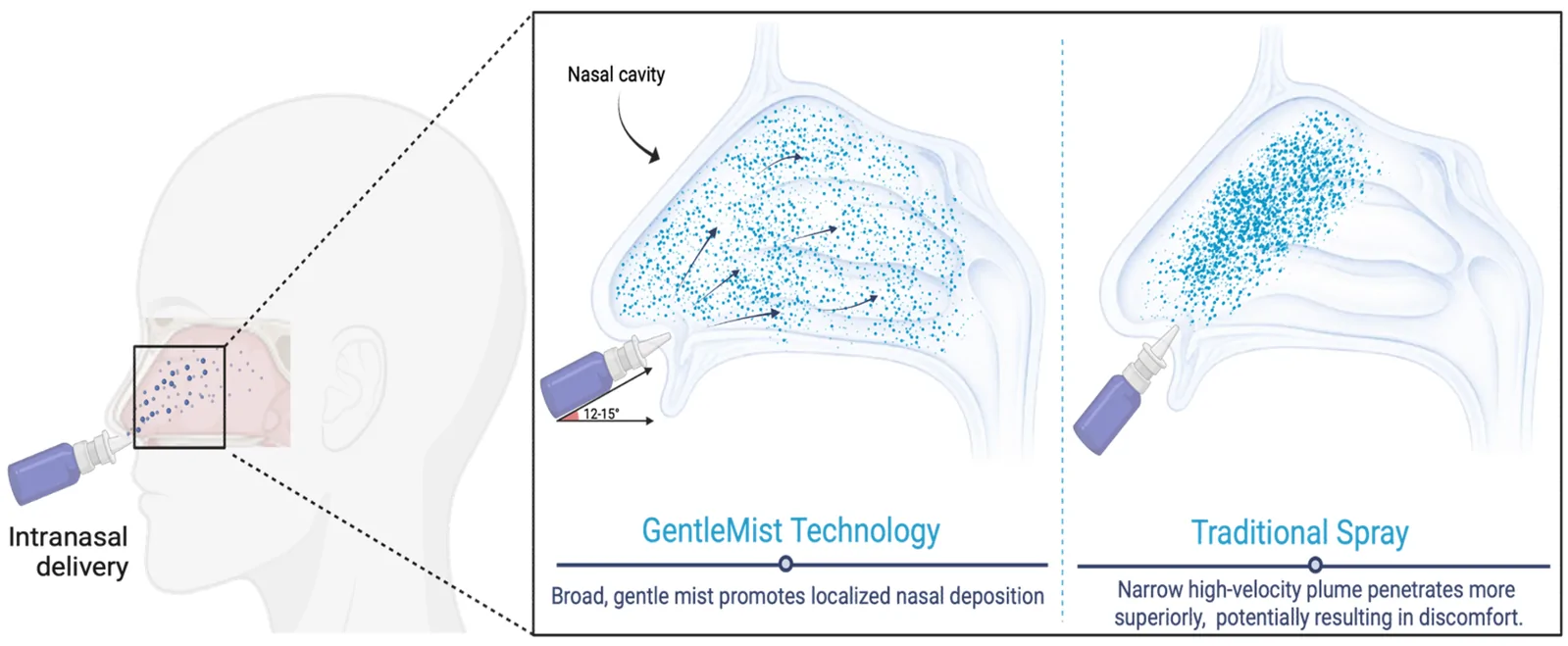
Conceptual comparison of intranasal plume geometry and distribution patterns. GentleMist Technology™ is depicted as generating a broader, more dispersed plume intended to support distributed mucosal coverage, whereas a conventional narrow-plume spray follows a more directional trajectory within the nasal cavity. This figure is a conceptual schematic intended to illustrate the design hypothesis; it is not drawn to scale and does not depict measured droplet-size distributions, spray velocities, or quantified deposition fractions. The comparative plume and deposition behaviour it illustrates has not yet been quantitatively demonstrated for this platform and remains to be established experimentally.

From a performance perspective, the principal proposed advantage is the generation of a finer and broader mist that may distribute medication more uniformly across the nasal mucosa as shown in Figure 2 [13,19,20]. This type of atomization is intended to reduce concentrated anterior impact and minimize excess runoff or dripping [19,20]. The platform is also described as supporting more appropriate dosing through angle-conscious administration, with the broader goal of improving therapeutic reach while maintaining comfort [13,19,20].

A second proposed advantage is potentially improved tolerability. GentleMist Technology™ is hypothesized to reduce impact pressure relative to conventional administration; this proposed effect has not yet been quantified for the platform against a defined comparator. Were such an effect confirmed, it would not be trivial in chronic or repetitive intranasal therapy, because a more tolerable administration experience may improve willingness to use the product repeatedly, particularly when repeated administration is required.

**Figure 3:**
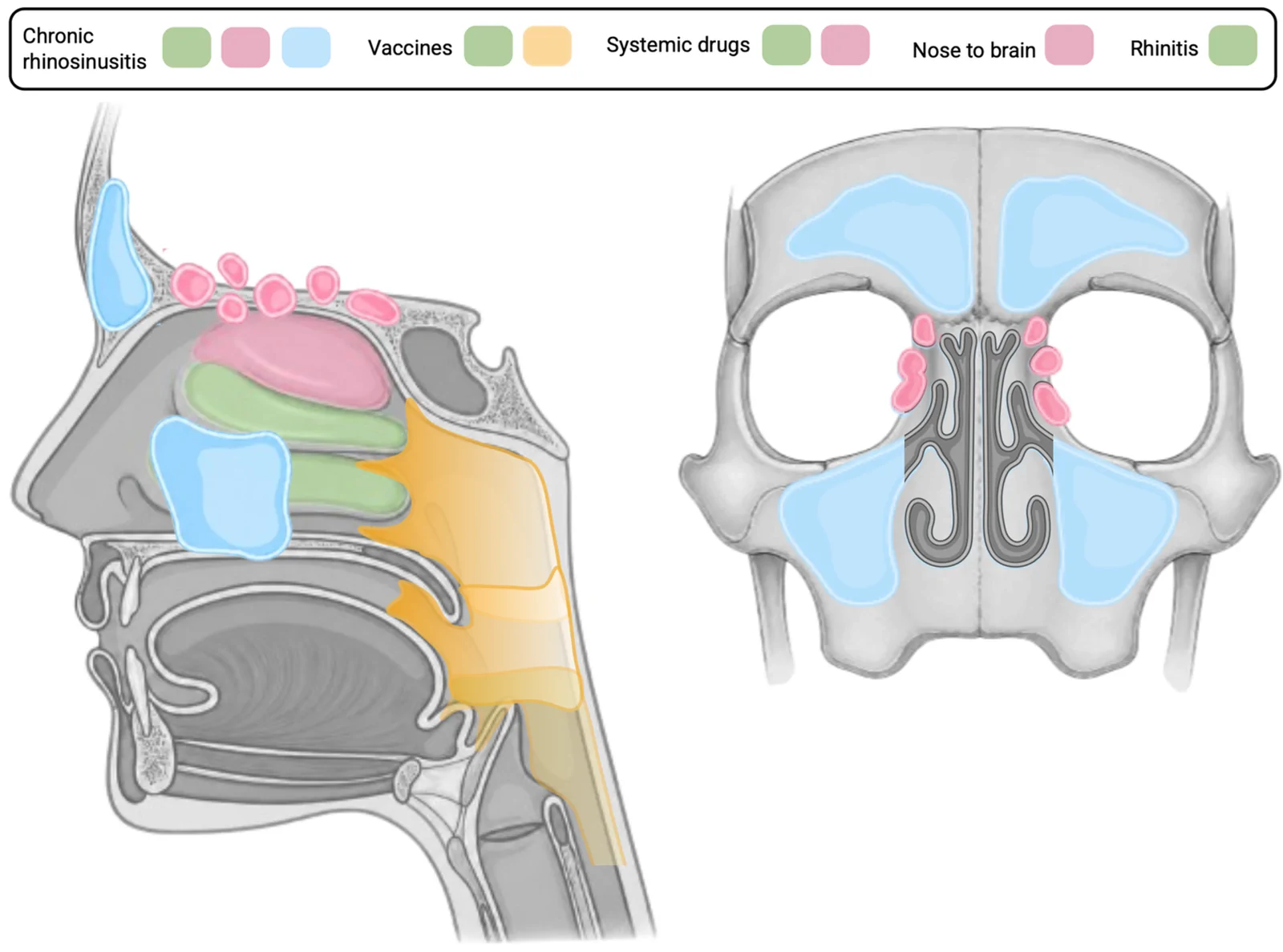
Schematic representation of nasal and paranasal anatomical regions relevant to local, systemic, and nose-to-brain drug delivery, highlighting potential target sites for different therapeutic applications.

Finally, current platform descriptions also frame GentleMist Technology™ as suitable for a broader class of intranasal applications beyond routine symptom relief. These include consumer-facing respiratory care, pediatric use, vaccine and biologics delivery, and situations in which gentle administration is especially desirable, such as in patients on oxygen or with irritated mucosa (Figure 3). These are proposed rather than demonstrated applications; to avoid equating them with clinically validated indications, they are classified by evidence status in Table 1. Taken together, these features indicate that the technology is being conceptualized not simply as a nozzle modification, but as a patient-centered intranasal interface intended to integrate comfort, targeting, and reproducibility.

### 5.2. Regional Targeting and Nose-to-Brain Delivery

A recurring theme of this review is that the nasal cavity should not be treated as a single delivery compartment. Its functional regions differ markedly in surface epithelium, accessibility to an incoming plume, and therapeutic relevance, and the region in which a dose is deposited more than the total quantity delivered, often determines the clinical outcome. Framing intranasal performance in terms of regional targeting therefore clarifies both the rationale for device engineering and the specific value proposition of an angle-optimized platform.

Four regions are of principal interest. The nasal vestibule and anterior segment are the most readily reached by conventional sprays but offer limited absorptive surface and are rapidly cleared, favoring local rather than systemic effect. The turbinate region presents a large, vascular, ciliated surface well suited to both local action and systemic absorption, but its convoluted geometry makes consistent deposition technique-dependent [14]. The nasopharynx, posterior to the turbinates, is relevant for conditions in which posterior mucosal exposure is desired and for agents intended to reach the oropharyngeal interface. The olfactory region, situated in the upper nasal space at the roof of the cavity, is anatomically small and difficult to access with conventional administration, yet it is the principal gateway for nose-to-brain delivery via the olfactory and trigeminal pathways [3].

Because these regions sit along different trajectories from the nostril, the geometry of administration, plume direction, velocity, and insertion angle is a direct lever on where a dose is deposited. Conventional sprays, typically administered at a steep angle with a narrow, high-velocity plume, tend to concentrate deposition anteriorly, with limited and variable penetration to posterior and superior regions. Angle-optimized administration is one strategy for redirecting deposition toward specific targets: in the feasibility work reviewed here, a shallower administration angle was associated with greater posterior nasopharyngeal penetration relative to a conventional angle [13]. This positions plume and angle control not merely as a comfort feature but as a mechanism for region-selective delivery.

The nose-to-brain application deserves particular caution. Reaching the olfactory region reproducibly is a recognized challenge, and even optimized sprays deliver only a small fraction of dose to the upper nasal space; demonstrating central delivery requires in vivo evidence that in vitro casts cannot provide. Accordingly, while the ability to modulate deposition region is conceptually relevant to CNS targeting, no nose-to-brain data exist for this platform, and any such application should be regarded as a hypothesis for future study rather than a demonstrated capability [15]. Regional targeting is best presented, then, as the unifying principle that links aerosol science, anatomy, and device design—one that GentleMist Technology™ illustrates for posterior/nasopharyngeal delivery and that remains to be tested for olfactory and nose-to-brain targets.

### 5.3. Formulation Compatibility and Device–Formulation Interactions

Intranasal therapies span diverse vehicle structures—aqueous solutions, suspensions, emulsions, and colloidal or nanoparticle dispersions—whose physicochemical properties interact directly with device mechanics. Rheological behaviour (viscosity and shear-thinning), surface tension, and, for disperse systems, particle size, polydispersity index (PDI), and zeta potential influence droplet-size distribution, plume angle, spray velocity, and the propensity for nozzle clogging [4,5,20]. Because the swirl chamber and spray orifice generate the mist by imparting shear and controlled break-up to the liquid, formulation viscosity and interfacial properties are expected to modulate the very plume characteristics on which the platform’s proposed advantages depend.

The available evidence for GentleMist Technology™, however, derives from aqueous, low-viscosity formulations (notably chlorpheniramine maleate with xylitol). Its compatibility with higher-viscosity or non-aqueous vehicles, emulsions, and colloidal or nanoparticle suspensions has not been reported, and whether the device can atomize such systems without altering droplet size, compromising dose uniformity, or clogging the orifice remains an open, testable question; suspended particulates and mucoadhesive or thixotropic excipients, in particular, may behave differently through a swirl-based nozzle than a simple solution. We therefore recommend that device–formulation compatibility be established experimentally across solutions, suspensions, emulsions, and colloidal dispersions, characterized by rheology, particle size, PDI, and zeta potential, and note that the patent and formulation literature on comparable swirl-chamber devices may indicate which vehicle classes are realistically deliverable; to our knowledge, current published and patent descriptions of the platform pertain to aqueous solution formulations, and its performance with suspensions, emulsions, and colloidal or nanoparticle dispersions has not yet been formally characterized.

## 6. Human Factors Engineering and the OTC Nasal Spray Paradigm

The increasing sophistication of intranasal devices makes human factors engineering central to development. FDA guidance for medical devices and combination products emphasizes that product performance depends not only on engineering reliability, but also on whether intended users can correctly perform essential tasks under realistic conditions [26,27,28]. For nasal sprays, these tasks may include shaking, priming, opening the cap, orienting the nozzle, maintaining head position, actuating correctly, cleaning, and storing the product properly.

This issue is particularly relevant in OTC settings. Unlike prescription products used under repeated clinician supervision, OTC sprays are often first used without demonstration, and they may be reused over long periods based on habit rather than instructions. Real-world evidence suggests that misuse of nasal medications is common [6], while interventional studies show that education can partially improve symptom outcomes in allergic rhinitis [18]. These observations suggest that usability is not a secondary feature but a determinant of real-world effectiveness.

For devices that rely on optimized angulation or nonstandard administration strategies, the stakes are even higher. A device with favorable bench-performance characteristics may still fail in practice if users cannot reproduce the intended technique. This is where human factors engineering becomes especially relevant for platforms such as GentleMist Technology™. If device performance depends on achieving a particular administration geometry or technique, then that technique must be understandable, teachable, and repeatable across intended users.

The recent literature on formulation factors and user experience also reinforces the need to integrate human-centered design into nasal product development [4]. User rejection may arise from bitterness, sourness, astringency, burning, or other negative sensations after intranasal use [4]. Therefore, for a platform intended to support repeated self-administration, patient experience must be treated as part of the therapeutic system, not merely as a subjective preference.

Evidence specific to GentleMist Technology™ comes from a published feasibility study evaluating an angle-optimized administration protocol in a CT-derived, 3D-printed nasal cast [13]. In that study, a 12–15° nozzle orientation produced greater posterior nasopharyngeal outflow per actuation than a conventional 67.5° orientation in both nostrils, with a markedly larger relative increase in the right nostril (right: 0.0649 vs. 0.0296 mL per actuation, *p* < 0.001; left: 0.0581 vs. 0.0505 mL, *p* = 0.019) [13]. Because these results were obtained in a single-donor anatomy, the 12–15° value should be interpreted as an anatomy-dependent, proof-of-concept finding rather than a universally optimized administration angle: septal deviation, turbinate geometry, vestibular morphology, and the natural nasal cycle differ between individuals and would be expected to shift the optimal orientation, and the larger right-nostril effect is itself most plausibly explained by such donor-specific asymmetry [9,11,14].

A companion human-factors evaluation included 191 lay users (50 simulated-use participants and 141 real-use participants). Essential steps—head positioning, nozzle angle, and spray delivery—met or exceeded a predefined 80% success threshold in both the simulated- and real-use cohorts, where task success was defined as correct completion of each step against the study’s prespecified criteria and the 80% threshold served as the predefined acceptance criterion for usability. The critical tasks, cohorts, success criterion, and observed use errors are summarized in Table 2. Detailed participant characteristics (age, sex, handedness, and prior nasal-spray experience) are reported in the source feasibility study [13]. The 80% acceptance threshold was adopted as a pragmatic usability-acceptance benchmark for essential tasks, consistent with human-factors validation practice under FDA guidance [26,27,28].

Priming was the exception: it was the lowest-performing step in the real-use cohort and fell below the acceptance threshold, primarily because participants with prior nasal-spray experience skipped it out of habit or overconfidence.

This last finding is arguably more informative than the success data because it identifies a specific, addressable design problem rather than a general usability claim. If accurate, it suggests that prior familiarity with conventional nasal sprays does not protect against, and may actively work against, correct use of a redesigned administration protocol—a counterintuitive but important consideration for OTC product design, where instructions are often written assuming that any prior nasal-spray experience is helpful rather than potentially misleading.

We emphasize that this feasibility study, although peer-reviewed and published, remains an early proof-of-concept investigation and should not be interpreted as definitive evidence [13]. Its findings are preliminary and hypothesis-generating, particularly pending independent replication across anatomically diverse models and clinical contexts (see Conflicts of Interest). In addition, the in vitro model lacked mucosal lining, mucociliary clearance, and dynamic breathing, and the usability arm did not include instrumented angle verification. Consequently, it remains uncertain how closely participants reproduced the intended administration angle. These limitations are discussed further in Section 8.

## 7. Translational and Clinical Implications

The translational value of a next-generation intranasal platform lies in the indications where comfort, deposition, and self-administration materially affect outcomes. Allergic rhinitis is a prime example. Intranasal therapies are among the mainstays of treatment, and the newest Allergic Rhinitis and its Impact on Asthma (ARIA)–European Academy of Allergy and Clinical Immunology (EAACI) recommendations continue to emphasize the central role of intranasal treatment strategies in allergic rhinitis care [29]. Allergic rhinitis is, however, a biologically heterogeneous condition, encompassing distinct phenotypes and inflammatory endotypes—including local allergic rhinitis and varying degrees of type 2 inflammation—so that mucosal state, secretion burden, and treatment response can differ substantially between patients [30]. This heterogeneity is directly relevant to a device rationale: baseline mucosal and secretory differences may influence deposition, retention, and tolerability, and a delivery strategy that performs well in one endotype may not generalize across the allergic-rhinitis population. In this setting, adherence, sensory burden, and ease of repeated use are highly relevant to effectiveness.

A second important translational area is targeted delivery to posterior nasal regions, the nasopharynx, or the upper nasal space. The upper nasal space has been identified as a promising region for systemic delivery, mucosal vaccination, and selected nose-to-brain approaches because regional deposition in that area may result in different absorption characteristics and residence time than lower-cavity delivery [3]. This makes device-mediated targeting clinically meaningful rather than merely mechanical.

A third area is viral respiratory disease and other conditions in which posterior or nasopharyngeal targeting may be desirable. While clinical outcomes ultimately depend on the active agent and indication-specific evidence, work on nasopharyngeal targeting and computationally guided administration suggests that intranasal devices can be intentionally developed around tissue-specific delivery objectives [7,8,21].

Early clinical evidence involving a chlorpheniramine maleate (CPM)-based intranasal formulation delivered via this platform lineage offers a preliminary, device-adjacent illustration of this rationale, though the evidence base remains limited in scale and requires substantial independent confirmation. A small, multicenter, randomized, double-blind pilot trial (n  =  29) found that intranasal CPM combined with xylitol produced a greater reduction in nasal symptoms than placebo over 30 days in patients with moderate-to-severe allergic rhinitis [22]. Separately, in vitro work reported virucidal activity of intranasally formulated CPM against SARS-CoV-2 [23], and a pooled analysis of 259 participants from two randomized, placebo-controlled outpatient COVID-19 trials found that patients who received intranasal CPM during acute infection were significantly less likely to develop persistent post-acute sequelae of SARS-CoV-2 (PASC) than those who received placebo [24]. These findings are notable, but they derive from a small pilot trial and a group of related studies conducted largely by overlapping investigators (see Conflicts of Interest). On their own, they do not establish a clinical benefit attributable specifically to the device or distinguish such a benefit from the pharmacologic activity of CPM. Larger, independently conducted trials are needed before device-specific clinical conclusions can be drawn.

In this context, the potential relevance of GentleMist Technology™ may extend beyond a single formulation. The platform illustrates how intranasal devices could be developed as integrated therapeutic interfaces designed to influence deposition, comfort, and user behavior simultaneously. Taken together, the available studies provide a scientific rationale and preliminary feasibility evidence for this platform concept. To keep these evidence types distinct, Table 3 appraises each contributing study by design, comparator, endpoint, and—critically—whether it provides device-specific, formulation-specific, or general evidence, and whether it was generated by developer-affiliated investigators. As that appraisal makes explicit, the chlorpheniramine-maleate findings are formulation-specific: they demonstrate pharmacologic activity of the active agent but do not, on their own, establish a clinical benefit attributable to the delivery device, and none of the device-specific evidence yet isolates the device contribution from the drug. Because these studies differ in design, endpoints, and level of device specificity, they should not be interpreted collectively as demonstrating a GentleMist-specific clinical benefit. Independent studies that distinguish device performance from formulation effects remain necessary.

## 8. Discussion

This review supports a central premise in contemporary intranasal drug delivery: the performance of a nasal product is not determined by formulation alone, but by the interaction among device mechanics, aerosol behavior, nasal anatomy, and user technique. In this framework, GentleMist Technology™ is best understood not simply as a modified spray actuator, but as an example of how engineering design, administration geometry, and human factors can be integrated to improve the translational value of intranasal drug delivery systems.

One of the most informative findings from the published proof-of-concept feasibility study summarized in Section 5 [13] is that small changes in nozzle angle may produce favorable differences in posterior nasopharyngeal penetration. The reported gain in the right nostril was substantially larger than in the left, plausibly related to donor-specific anatomic asymmetry, including septal deviation. This observation, drawn from a single donor cast a recently published, peer-reviewed study, is nonetheless directionally consistent with the broader CFD literature showing that vestibular morphology, turbinate geometry, septal configuration, and even the natural nasal cycle can meaningfully alter local airflow and spray deposition [8,11,12].

GentleMist Technology™ should not be framed as providing a universally optimal administration angle for all users, but rather as an illustration that device performance may be enhanced when spray mechanics and administration instructions are developed with anatomy-sensitive principles in mind, a hypothesis that now requires testing across anatomically diverse models before it can be treated as established.

A second point raised by that work is that it moved beyond fluid mechanics alone to ask whether optimized delivery can realistically be performed by lay users—a question that is central for OTC products, where theoretical device advantages are meaningless if untrained users cannot reproduce the intended technique. The reported finding that essential steps met a predefined success threshold in both simulated and real-use conditions is encouraging as a proof of concept, though that evidence still comes from a single-site proof-of-concept.

The reported failure point, priming being disproportionately skipped by experienced users, is arguably more informative than the success data, because it identifies a specific, addressable design problem rather than a general claim of usability. If accurate, it would suggest that prior familiarity with conventional nasal sprays does not protect against, and may actively work against, correct use of a redesigned administration protocol. For OTC platforms that depend on a specific technique, this reframes priming, and similar setup steps, as a labeling and device-affordance problem rather than a training problem: instructions that assume a blank-slate user, rather than one anchored to prior habits, may be necessary.

**Figure 4:**
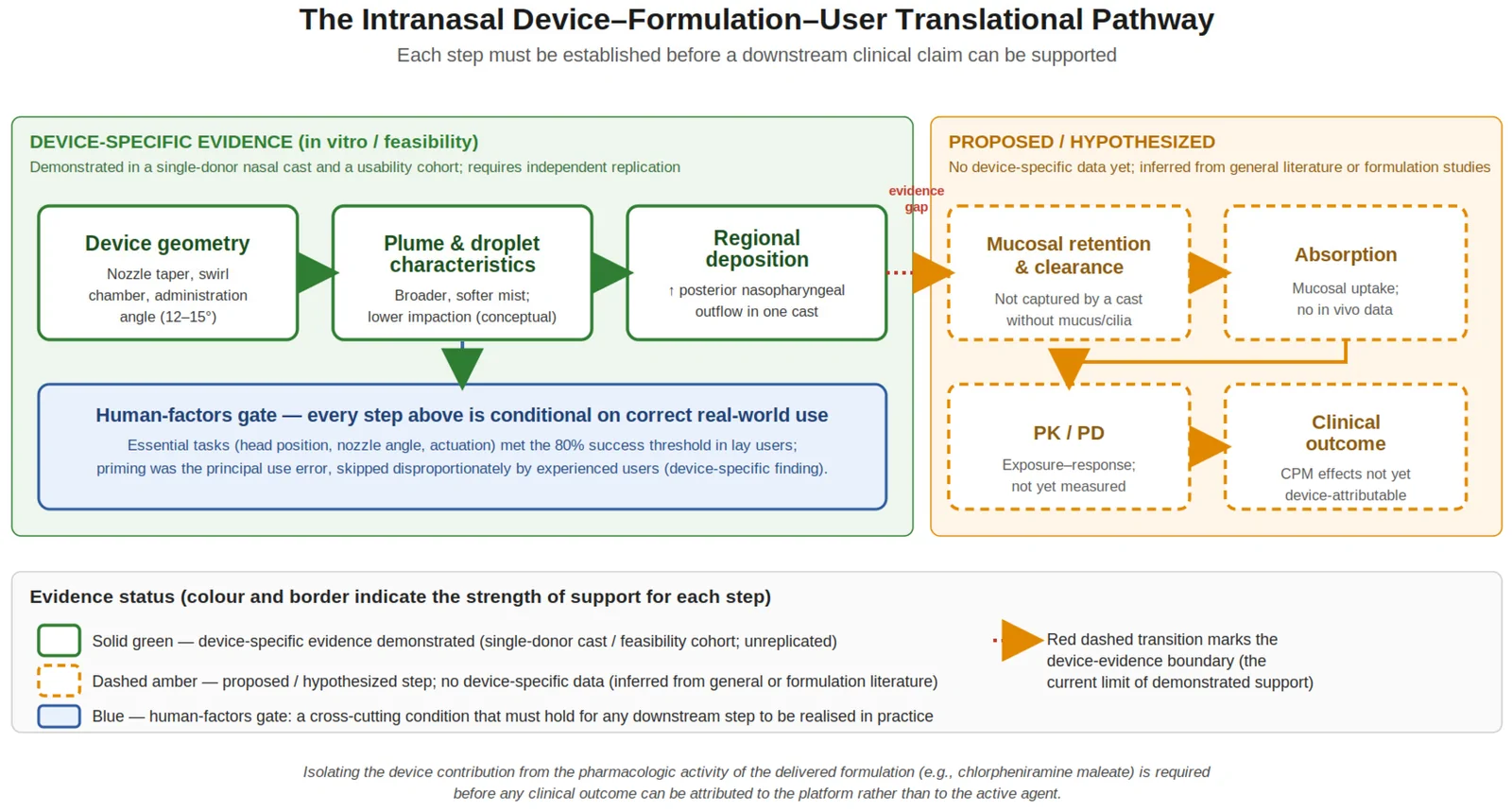
The intranasal device–formulation–user translational pathway. Device geometry shapes plume and droplet characteristics, which determine regional deposition; deposition must then be retained, absorbed, and translated through pharmacokinetic/pharmacodynamic (PK/PD) effects into a clinical outcome. Solid green steps denote device-specific evidence demonstrated in a single-donor nasal cast and a usability cohort (unreplicated); dashed amber steps are proposed or hypothesized, with no device-specific data. The human-factors gate (blue) is a cross-cutting condition: every downstream step depends on correct real-world use. The red dashed transition marks the current boundary of demonstrated support, beyond which isolating the device contribution from the pharmacologic activity of the delivered formulation (e.g., chlorpheniramine maleate) is required before any clinical outcome can be attributed to the platform.

These observations, taken together with the broader literature reviewed in Section 3, Section 4 and Section 5, support treating the nasal spray as an integrated therapeutic interface rather than a container for a formulation. Droplet size distribution, plume structure, and actuation mechanics are not separable from user posture, nozzle angle, insertion depth, sensory burden, and instruction adherence; the clinical result reflects the device–formulation–user system as a whole, not the formulation in isolation (Figure 4).

The current evidence base, however, defines the boundaries of what can be concluded. Beyond the limitations of the in vitro model itself (absence of mucosal lining, mucociliary clearance, dynamic breathing, and patient-specific biologic variability), the central feasibility data discussed here derive from a recently published, single-center proof-of-concept study that remains unreplicated. This is a material limitation, not a minor caveat, and readers should weight the corresponding claims accordingly until independent replication and peer review are available. Similarly, the clinical evidence summarized in Section 6—while independently useful—derives substantially from a related, overlapping group of investigators and has not yet been extended to larger, independently conducted confirmatory trials that could isolate device-attributable effects from the pharmacologic activity of CPM (see Conflicts of Interest).

Accordingly, the next step for technologies such as GentleMist Technology™ is not simply larger usability studies, but integrated, independently verifiable translational validation: anatomically diverse 3D models, rheologically characterized formulations, simulated breathing, objective (instrumented) angle measurement, and pharmacokinetic or pharmacodynamic endpoints capable of linking deposition to mucosal exposure and therapeutic effect—ideally conducted, or independently replicated, by investigators without a financial interest in the outcome. Stratified human factors analyses by age, handedness, prior nasal spray experience, and symptom burden would also be valuable, since OTC nasal products are used by highly heterogeneous populations.

Taken together, the literature reviewed here, and the preliminary feasibility data discussed with appropriate caution, support treating GentleMist Technology™ as a model of a broader shift in intranasal product development: from dispensing a dose toward engineering a reproducible, anatomy-informed, and human-factors-validated therapeutic interaction. Whether this specific platform achieves that goal is an empirical question that the current evidence base does not yet answer, and that independently replicated, peer-reviewed research should address.

## 9. Limitations and Future Directions

The evidence assembled in this review supports a conceptual argument that intranasal performance is best understood as an integrated device–formulation–user problem more strongly than it supports any specific quantitative claim about GentleMist Technology™. Several limitations bound what can currently be concluded and should be read as constraints on interpretation rather than as incidental caveats.

First, the central device-specific evidence derives from a single, recently published proof-of-concept study conducted in a single-donor anatomical cast and a single usability cohort [13]. Deposition outcomes obtained in one nasal geometry may not generalize across the anatomical variability, vestibular morphology, turbinate geometry, septal configuration, and the nasal cycle—that this review itself identifies as a dominant determinant of intranasal delivery [13,14,15,25]. Second, the in vitro model lacked a mucosal lining, mucociliary clearance, and dynamic airflow, so it cannot capture residence time, absorption, or the clearance-driven loss of dose that governs real-world exposure. Third, and most importantly for interpretation, the available data do not separate the contribution of the delivery device from the intrinsic pharmacologic activity of the chlorpheniramine maleate (CPM) formulation delivered through it; improved outcomes in the CPM studies [22,23,24] cannot presently be attributed to the platform rather than to the active moiety. Finally, a meaningful proportion of the primary evidence originates from investigators and sources affiliated with the technology’s developer; this is disclosed in full (see Conflicts of Interest) but nonetheless warrants cautious, independent appraisal.

These limitations map directly onto a tractable research agenda. The most immediate priority is independent replication of the deposition findings across anatomically diverse, multi-donor casts and, ultimately, in vivo confirmation for example by gamma scintigraphy so that region-specific targeting claims can be tested rather than inferred. In parallel, studies designed to isolate the device effect from the formulation effect (for instance, delivering an identical formulation through the platform and a conventional comparator) would establish whether the platform confers benefit independent of CPM.

Beyond validation, the framework advanced here points toward formulation–device co-optimization—matching droplet size, viscosity, and mucoadhesive residence time to the intended deposition region—and toward controlled benchmarking against established predicate devices. Extending evaluation across indications (allergic rhinitis, viral upper respiratory illness, and, more speculatively, olfactory-region targeting for nose-to-brain applications) would clarify where an integrated device–formulation–user approach adds the most value. Each limitation above maps onto a specific study design: the single-donor constraint onto multi-donor and anatomically diverse casts; the absence of mucosa and airflow onto physiologically representative models with simulated breathing; the lack of instrumented angle verification onto objective angle measurement; and the inability to separate device from drug onto head-to-head studies delivering an identical formulation through the platform and a predicate device. Until such data are available, GentleMist Technology™ is most appropriately positioned as an illustrative example of an emerging design philosophy rather than as a validated performance advance.

## 10. Conclusions

Intranasal drug delivery is increasingly understood as a product system in which formulation properties, device characteristics, nasal anatomy, and user technique jointly influence performance. This perspective is particularly relevant in over-the-counter settings, where administration commonly occurs without direct professional supervision and product performance depends on the ability of intended users to complete essential administration tasks consistently.

Within this framework, GentleMist Technology™ provides an illustrative example of a device-centered development approach that integrates plume design, administration geometry, and human-factors evaluation. In a single-donor anatomical model, a shallower administration angle was associated with greater posterior nasopharyngeal outflow than the comparator angle. In the accompanying usability evaluation, most essential tasks met the predefined success criterion, whereas priming was the principal observed use error. These findings support technical and usability feasibility but do not establish superior regional deposition across diverse anatomies, improved tolerability or adherence, or clinical effectiveness. Clinical studies of intranasal chlorpheniramine provide formulation-specific context but do not isolate the contribution of the delivery device.

Further evaluation should include quantitative spray characterization, anatomically diverse and physiologically representative models, objective measurement of administration technique, and direct comparison with defined nasal spray devices. Clinical studies should be designed to distinguish device effects from formulation effects and, where appropriate, relate regional deposition to pharmacokinetic, pharmacodynamic, or patient-relevant outcomes. Until such evidence is independently replicated, GentleMist Technology™ should be regarded as an early example of an integrated intranasal development strategy rather than as an established performance advance.

## Figures and Tables

**Table 1:** Comparative appraisal of the principal evidence discussed in this review, distinguishing device-specific, formulation-specific, and general intranasal-delivery evidence and indicating developer affiliation.

Study [Ref]	Design, Model/Population	Comparator	Main Endpoint and Key Finding	Evidence Category	Developer-Affiliated	Key Limitation
Alas-Pineda et al., 2026 [13]	In vitro nozzle-angle deposition in a single-donor, CT-derived 3D-printed nasal cast; paired usability study in 191 lay users (50 simulated-, 141 real-use)	Conventional 67.5° angle; 80% task-success threshold	Posterior nasopharyngeal outflow and task success: 12–15° gave greater posterior outflow (right 0.0649 vs. 0.0296 mL, *p* < 0.001; left 0.0581 vs. 0.0505 mL, *p* = 0.019); global task completion 90.8% (simulated)/86.1% (real-use); priming below threshold	GentleMist-specific (device)	Yes	Single donor; no mucosa, clearance, or dynamic airflow; no instrumented angle
Basu et al., 2022 [21]	Patient-experience study of a computationally guided administration protocol	Shallower vs. more vertical angle	Comfort and perceived penetration: shallower angle rated more comfortable and more favorable for penetration	Device-adjacent (protocol)	Yes	Subjective endpoints; no controlled deposition measurement
Sanchez-Gonzalez et al., 2021 [22]	Multicenter, randomized, double-blind pilot trial; intranasal CPM + xylitol in moderate-to-severe allergic rhinitis; *n* = 29	Placebo over 30 days	Nasal symptom reduction: greater with intranasal CPM + xylitol than placebo	Formulation-specific (not device-isolating)	Yes	Very small; effect attributable to formulation, not device
Westover et al., 2020 [23]	In vitro virucidal assay of intranasally formulated CPM against SARS-CoV-2	Assay controls	Virucidal activity reported in vitro	Formulation-specific (not device-isolating)	Yes	In vitro only; no device or clinical data
Valerio-Pascua et al., 2024 [24]	Pooled analysis of two randomized, placebo-controlled outpatient COVID-19 trials; intranasal CPM; *n* = 259	Placebo during acute infection	PASC incidence: lower likelihood with intranasal CPM than placebo	Formulation-specific (not device-isolating)	Yes	Overlapping investigators; formulation, not device, effect
Independent CFD/in vitro studies [7,8,9,10,11,25]	Computational fluid dynamics and 3D-printed nasal-cast studies of nozzle design, angle, vestibular anatomy, and the nasal cycle	Varied nozzles, angles, anatomies	Regional deposition: geometry and administration angle significantly alter regional/nasopharyngeal deposition	General intranasal-delivery evidence (not GentleMist)	No	Model-based; not platform-specific

CPM, chlorpheniramine maleate; CFD, computational fluid dynamics; PASC, post-acute sequelae of SARS-CoV-2 infection. “Developer-affiliated” denotes authorship or funding overlap with the developer of GentleMist Technology™ (see Conflicts of Interest).

**Table 2:** Critical user tasks assessed in the GentleMist Technology™ human-factors feasibility evaluation, with the tested cohorts, the predefined success criterion, and the observed result or use error [13].

Essential User Task	Cohort Assessed	Predefined Success Criterion	Observed Result/Use Error
Head positioning	Simulated-use (n = 50) and real-use (n = 141) lay users	≥80% of participants completing the step correctly	Above the ≥80% threshold in both cohorts
Nozzle angle/orientation	Simulated- and real-use lay users	≥80% correct	Above the ≥80% threshold in both cohorts
Spray actuation/delivery	Simulated- and real-use lay users	≥80% correct	Above the ≥80% threshold in both cohorts
Priming before use	Simulated- and real-use lay users	≥80% correct	Below the ≥80% threshold in the real-use cohort—the principal observed use error, driven by experienced users skipping priming
Remaining steps of the nine-step application protocol (e.g., shake device; remove dust cap; insert nozzle; occlude the opposite nostril; actuate while inhaling gently; repeat in the contralateral nostril; replace cap)	—	≥80% correct	Above the ≥80% threshold; no step other than priming fell below the criterion

The source study [13] reported global mean task-completion rates of 90.8 ± 8.2% (simulated-use) and 86.1 ± 11.1% (real-use), both above the predefined ≥80% acceptance threshold, with priming the step that fell below threshold in the real-use cohort [13].

**Table 3:** Proposed applications of GentleMist Technology™ classified by evidence status, to distinguish demonstrated capability from preliminary, indirect, and future/hypothetical applications.

Proposed Application	Evidence Status	Current Basis	Evidence Needed to Advance
Allergic rhinitis (symptom relief)	Preliminary—formulation-specific	Small pilot RCT of intranasal CPM + xylitol vs. placebo [22]	Larger, independent, device-isolating trials
Posterior/nasopharyngeal targeting	Preliminary—device-adjacent	Single-donor feasibility cast [13]; supportive CFD [7,8]	Independent multi-donor and in vivo (e.g., scintigraphy) confirmation
Viral upper-respiratory illness (COVID-19/PASC)	Preliminary—formulation-specific	In vitro virucidal data [23]; pooled CPM outcome data [24]	Trials isolating device from CPM pharmacology
Pediatric use	Future/hypothetical	No device-specific data; general suitability proposed	Dedicated pediatric feasibility and safety studies
Vaccine and biologics delivery	Future/hypothetical	Indirect—the general upper-nasal-space literature [3]	Platform-specific deposition and immunogenicity studies
Gentle administration (oxygen-dependent/irritated mucosa)	Future/hypothetical	Proposed comfort rationale; no comparative tolerability data	Controlled comparative tolerability studies
Nose-to-brain/olfactory-region delivery	Future/hypothetical	Indirect—the general olfactory-deposition literature [3,15,16]; no platform data	In vivo olfactory-deposition and CNS-exposure evidence

Evidence-status categories: demonstrated (device-specific, replicated); preliminary (single study or formulation-specific); indirect (the general literature, not platform-specific); future/hypothetical (proposed, no supporting platform data).

## Data Availability

No new data were created or analyzed in this study. Data sharing is not applicable to this article.

## Supplementary Materials

The following supporting information can be downloaded at: https://www.mdpi.com/article/10.3390/pharmaceutics18091128/s1, Figure S1: Study-Selection Flow Diagram.

## Abbreviations

The following abbreviations are used in this manuscript:

CPM	Chlorpheniramine maleate
CFD	Computational fluid dynamics
OTC	Over-the-counter
ARIA	Allergic Rhinitis and its Impact on Asthma
EAACI	European Academy of Allergy and Clinical Immunology
FDA	U.S. Food and Drug Administration
PASC	Post-acute sequelae of SARS-CoV-2 infection
PD	Pharmacodynamic(s)
PDI	Polydispersity index
PK	Pharmacokinetic(s)
SANRA	Scale for the Assessment of Narrative Review Articles
SARS-CoV-2	Severe acute respiratory syndrome coronavirus 2
